# Real-time broadband terahertz spectroscopic imaging by using a high-sensitivity terahertz camera

**DOI:** 10.1038/srep42540

**Published:** 2017-02-15

**Authors:** Natsuki Kanda, Kuniaki Konishi, Natsuki Nemoto, Katsumi Midorikawa, Makoto Kuwata-Gonokami

**Affiliations:** 1 RIKEN Center for Advanced Photonics, RIKEN, 2-1 Hirosawa, Wako, Saitama 351-0198, Japan; 2 Photon Science Center, The University of Tokyo, 7-3-1 Hongo, Bunkyo-ku, Tokyo 113-8656, Japan; 3 Institute for Photon Science and Technology, The University of Tokyo, 7-3-1 Hongo, Bunkyo-ku, Tokyo 113-0033, Japan; 4 Department of Physics, The University of Tokyo, 7-3-1 Hongo, Bunkyo-ku, Tokyo 113-0033, Japan

## Abstract

Terahertz (THz) imaging has a strong potential for applications because many molecules have fingerprint spectra in this frequency region. Spectroscopic imaging in the THz region is a promising technique to fully exploit this characteristic. However, the performance of conventional techniques is restricted by the requirement of multidimensional scanning, which implies an image data acquisition time of several minutes. In this study, we propose and demonstrate a novel broadband THz spectroscopic imaging method that enables real-time image acquisition using a high-sensitivity THz camera. By exploiting the two-dimensionality of the detector, a broadband multi-channel spectrometer near 1 THz was constructed with a reflection type diffraction grating and a high-power THz source. To demonstrate the advantages of the developed technique, we performed molecule-specific imaging and high-speed acquisition of two-dimensional (2D) images. Two different sugar molecules (lactose and D-fructose) were identified with fingerprint spectra, and their distributions in one-dimensional space were obtained at a fast video rate (15 frames per second). Combined with the one-dimensional (1D) mechanical scanning of the sample, two-dimensional molecule-specific images can be obtained only in a few seconds. Our method can be applied in various important fields such as security and biomedicine.

Spectroscopic imaging is a combination of spectroscopy and imaging that provides more information regarding the spectra of a sample than conventional imaging. For example, it enables the visualization of the spatial distribution of different materials based on their spectral information and is widely applied in biomedicine, agriculture, and industry^1^. Spectroscopic imaging is sophisticated owing to the development of imaging devices, e.g., charge coupled device (CCD) cameras, in the visible spectral region.

Terahertz (THz) imaging has strong practical applications because opaque materials in other frequency region (visible or near infrared) are often transparent in the THz region, for example papers and plastics^2^,^3^. In addition, spectroscopic imaging in the THz spectral region is important for substance identification because many elementary excitations in solids (phonons and magnons) and gases (molecular vibrations and rotations) exist in this region. By utilizing the THz responses of these low-energy modes and delocalized vibrations, THz spectroscopic imaging enables non-contact and label-free molecule-specific imaging^4^,^5^,^6^. However, the THz frequency region still has limitations in light sources, detectors and optical components compared to other frequency regions despite its importance.

In conventional THz imaging systems, capturing one image may require several minutes because it involves beam (or sample) scanning with a single detector. In addition, frequency scanning needs to be performed by either tuning a continuous wave (CW) THz source or by moving the time-delay stage in the THz time-domain spectroscopy (TDS). In both cases, obtaining one frame in THz spectroscopic imaging requires 2D scanning of the THz beam over the sample surface as well as scanning of the beam frequency. Such a three-dimensional (3D) scanning system is based on mechanical motions that drastically reduce the data acquisition rate and hamper real-time imaging.

To improve the data acquisition rate, several methods have been proposed in both TDS- and CW-based systems. In TDS, spatial scanning can be avoided via focal-plane imaging with large aperture electro-optic (EO) crystals and CCD cameras, which is called 2D electro-optic (EO) sampling^6^,^7^,^8^. Methods have also been developed to avoid scanning time delays, e.g., asynchronous optical sampling^9^, chirped pulsed gating^10^, and using echelons for the probe beam^11^. However, it is difficult to combine these methods and 2D EO sampling. In CW-based systems, the use of arrayed detectors or cameras^12^,^13^ is one method to avoid sample scanning in THz spectroscopic imaging, in contrast to conventional methods^4^,^14^,^15^; however, frequency scanning is still necessary. In addition, the lack of a tunable high-power CW THz source and the low sensitivity of THz cameras make real-time spectroscopic imaging difficult with CW-based methods.

Recent developments in THz sources and detectors offer us another approach to real-time spectroscopic imaging. Regarding THz sources, the generation of intense THz pulses has been established via optical rectification in LiNbO_3_ crystals with the tilted pulse excitation method^16^, which allows THz pulses to be obtained with a peak electric field strength of 1 MV/cm using a table-top scale laser system^17^. This efficient THz generation method is suitable for spectroscopic imaging because large average power and a broadband spectrum can be obtained. With regards to THz detectors, 2D arrayed uncooled detectors have recently been developed by using micro bolometers^18^,^19^ and CMOS techniques^20^, which make it possible to obtain 2D movies at video-rate. In particular, we have recently reported the development of a high-sensitivity THz camera with a sensitivity of approximately 100 pW/pix at 1 THz^21^.

The combination of a broadband THz source and a high-sensitivity THz camera permits the design of a multi-channel diffraction spectrometer, as shown in Fig. 1(a), which is a common method in the visual and IR spectral ranges^22^. Specifically, in the 2D detector array, one axis can be used to measure the frequency, while the other can be used for the spatial coordinate. Even though far-infrared diffraction spectrometers were developed in the 1950 s^23^,^24^, they were difficult to apply to spectroscopic imaging because the light source was heat-based and the detector was a single bolometer or Golay cell sensor. Very recently, the development of a multi-channel THz spectrometer with a THz quantum cascade laser and an infrared camera has been reported^25^. This spectrometer has a high frequency resolution and can be employed for gas analysis; however, its limited tunability, insufficient average power, and low sensitivity infrared camera do not allow it to be employed for broadband spectroscopic imaging in the 1 THz spectral range.

In this study, we report a broadband THz spectroscopic imaging system based on a high-sensitivity uncooled THz camera. To take an advantage of the two-dimensionality of the THz camera, we constructed a multi-channel spectrometer with a diffraction grating and high-power THz source. With sugar molecules as test samples, we demonstrate real-time molecule-specific THz imaging in 1D and high-speed THz imaging of the 2D molecule distributions.

## Results

### Multi-channel spectrometer

**Figure 1:**
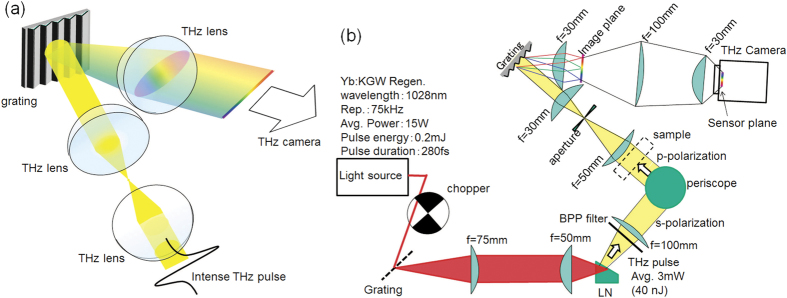
Schematic of the experimental setup. (**a**) Schematic of the real-time multi-channel spectrometer with diffraction grating. (**b**) Setup for the experiment. A high power THz pulse source and a high-sensitivity THz camera were used.

**Figure 2:**
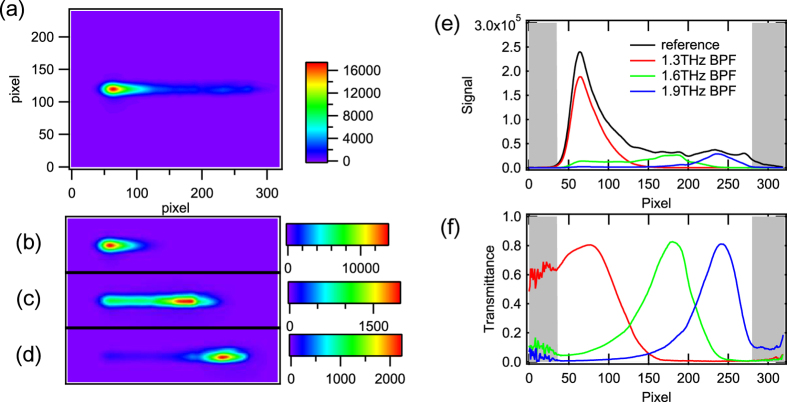
Results of the THz multi-channel spectrometer. Frequency-resolved profiles of the THz beams obtained using the THz camera (**a**) without any sample and with bandpass filters whose center frequency were (**b**) 1.3 THz, (**c**) 1.6 THz, and (**d**) 1.9 THz. (**e**) Horizontal profile of the camera images integrated in the vertical direction. (**f**) Transmittance spectra of the bandpass filters normalized to the reference profile.

As mentioned above, we developed a diffraction type multi-channel spectrometer with a high-power THz source and a high-sensitivity THz camera, as shown in Fig. 1(b) (see “Experimental setup” in “Methods” for details). Figure 2(a) shows the image obtained by the THz spectrometer without a sample. A horizontal line-shaped profile is observed, where the left side corresponds to the lower frequency components, and vice versa. Figure 2(b–d) show the images obtained for the band pass filters (BP-13 N1, BP-16 N1, and BP-19M, CDP corp.) with center frequencies of 1.3 THz, 1.6 THz, and 1.9 THz, respectively, which are inserted into the “sample” position indicated in Fig. 1(b). The bright position of the profile for each filter clearly depends on the center frequency of the filter. Figure 2(e) indicates the signal intensities that were obtained by integrating the images shown in Fig. 2(b–d) in the vertical direction. By normalizing them to the reference spectrum, the transmittance spectra of the test samples were obtained (Fig. 2(f)). As depicted by the shadows in Fig. 2(f), both side edges of the profile should be ignored due to small signal to noise ratio (SNR) caused by the limitation of the aperture sizes of the lenses. Note that the calculation of the line profiles and normalizations were performed in real-time using a commercially available computer. Therefore, the maximum frame rate of this measurement system was 15 frames per second (fps).

The horizontal axes in Fig. 2 are pixels; therefore, a calibration from the pixel number (*p*) to the frequency (*f*) is necessary to use this system as a spectrometer. Such a calibration is usually performed by comparing a spectra obtained by the spectrometer with an already-known spectra of the same material from a database. Here, we used the absorption lines of water vapor for the calibration.

The absorption line data are available in a database distributed by NASA^26^. For comparison with the obtained data, we measured the transmittance spectrum *T(p*) of the water vapor by taking the ratio of the spectra with and without dry air purging the setup, where *p* is the pixel position of the THz camera. The relationship between the pixel position and the frequency of the THz wave essentially satisfies the equation of diffraction. In addition, the distortion of the image from aberration and the frequency-dependent frequency resolution were taken into account. By fitting the measured spectrum into the one in the database, the calibration curve, *f(p*), was obtained. More details are included in “Calibration of the frequency” in “Methods.”

**Figure 3:**
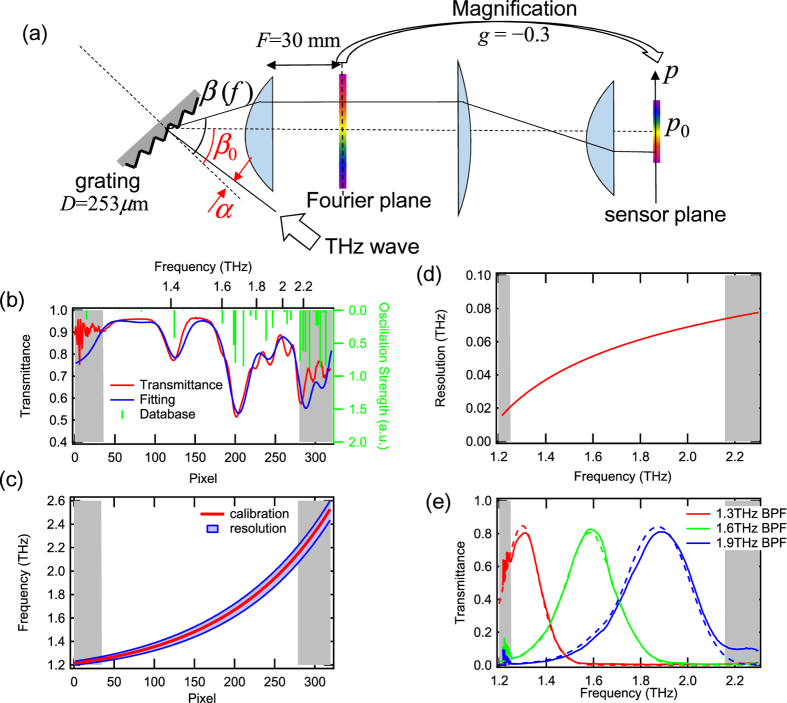
Calibration of the frequency from the pixels. (**a**) The geometrical relationship between the diffraction angle *β(f*) and the focused position *p(f*). Other geometrical parameters are also defined. (**b**) Transmission spectra of water vapor in air. The red curve is the result from the multi-channel spectrometer, the blue curve is the fit with the database, and the green curve is the absorption line in the database. The shadows on both sides show the region that is unreliable due to the scarcity of signals. (**c**) The calibration curve from pixel space to frequency space in the spectrometer. The light blue area shows the frequency resolution. (**d**) The frequency-dependent frequency resolution, which corresponds to the width of the blue area in (**c**). (**e**) The transmission spectra of the bandpass filters obtained using the multi-channel spectrometer (solid) and TDS (dashed). The TDS spectra are convolved with the frequency resolution shown in (**d**).

Figure 3(b) shows the result of the fitting. The absorption lines near 1.4 THz and 1.7 THz are well reproduced. The calibration curve obtained from the fitting parameters is shown in Fig. 3(c). From this calibration analysis, the frequency resolution is also evaluated, which depends on the frequency as shown in Fig. 3(d). The frequency resolution is approximately 0.03 THz at 1.3 THz, and becomes broader at higher frequency, e.g. 0.07 THz at 2.1 THz.

To confirm the validity of the calibration, the horizontal axis of Fig. 2(f) was converted to frequency using the curve in Fig. 3(c). In Fig. 3(e), the spectra from this spectrometer and those from the TDS-based setup are compared. The spectra from TDS are convolved with the frequency resolution, which is shown in Fig. 3(d). The spectra obtained by the spectrometer and TDS show good agreement.

**Figure 4:**
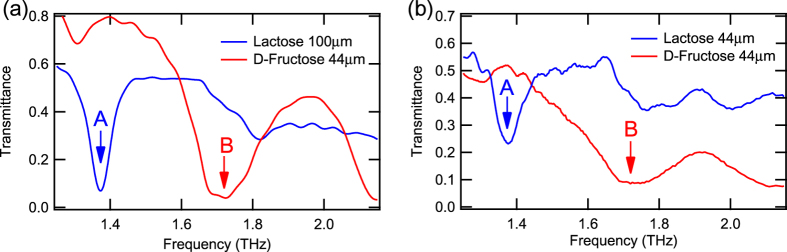
The fingerprint spectra of sugar samples. Trasmission spectra of lactose and D-fructose obtained using (**a**) TDS and (**b**) the multi-channel spectrometer. The characteristic absorption peaks are indicated by the arrows at (A) 1.37 THz for lactose and (B) 1.72 THz for D-fructose.

Next, we demonstrate the real-time observation of the fingerprint spectra of molecules using the presented THz spectrometer. We used lactose and D-fructose as test samples (See “Methods” for details on sample preparation). The transmission spectra from the TDS and the camera spectrometer are shown in Fig. 4(a) and (b), respectively. In both methods, the absorptions at 1.37 THz for lactose and at 1.72 THz for D-fructose are clearly observed, and these different features of the spectra enable these molecules to be distinguished.

### Spectroscopic imaging

**Figure 5:**
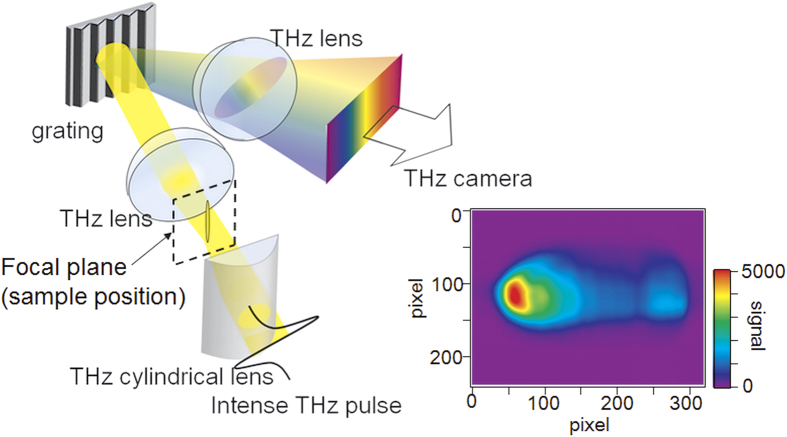
Schematic of the imaging spectroscopy. The inset shows the reference image.

The presented THz spectrometer can be applied to spectroscopic imaging using the vertical axis for spatial 1D imaging while the horizontal axis is retained for spectroscopy. In Fig. 1, the THz beam is focused on a circular spot with an aspherical convex lens at the entrance aperture of the spectrometer. By exchanging that lens with a cylindrical one, the THz beam is focused just in the horizontal direction and has a line-shaped profile at the entrance, as shown in Fig. 5. In this case, the image of the focal position in the vertical direction is transferred by the lens pair, while the horizontal direction corresponds to the frequency. Note that the aperture at the entrance of the spectrometer is removed to place the sample at the focal plane, as shown in Fig. 5, unlike in the case of Fig. 1(b). The frequency resolution does not worsen because the horizontal focusing size is sufficiently small. The reference image, which is obtained without any sample, is also shown in Fig. 5. The image is vertically expanded compared to the image shown in Fig. 2(a).

**Figure 6:**
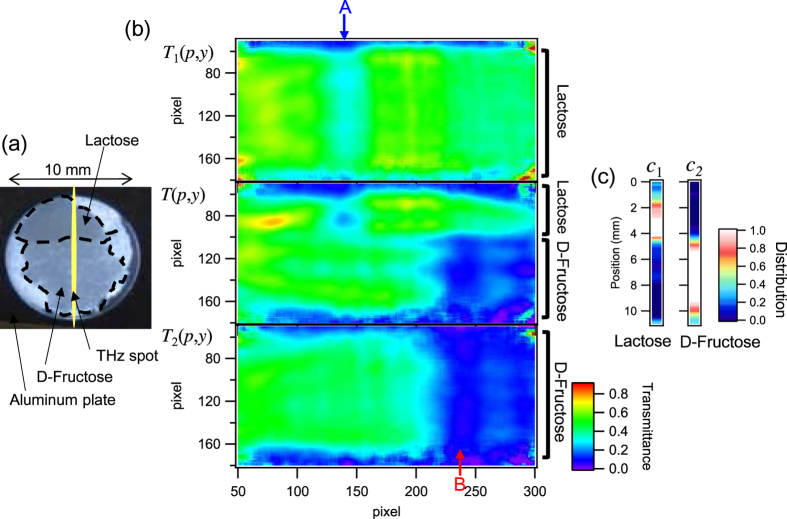
Results of the spectroscopic imaging and the independent component analysis. (**a**) Optical image of the test sample with the lactose and D-fructose domains. (**b**) Transmittance image of the test sample (middle), and the counterparts for the pure lactose (top) and D-fructose (bottom) samples, respectively. The absorption lines shown in Fig. 4 are again indicated with arrows and characters (A and B). (**c**) The distribution of each component obtained from the analysis.

Using this scheme, molecule-specific imaging can be performed. Figure 6(a) shows an optical image of a test sample that has two domains of lactose and D-fructose. The thickness of the sample is 44 μm. The domains and the boundaries can be seen as indicated by the dashed line in Fig. 6(a) because of the small difference in the absorption or scattering in the visible region between the two materials. White areas at both sides of the sample are empty. The line-shaped THz beam shines on the sample and includes the domain boundary. Taking the ratio of images, such as shown in Fig. 5, with and without a sample, a transmission spectral image *T(p, y*) can be obtained as a function of the horizontal pixel *p* and the vertical position *y*, as shown in Fig. 6(b) middle. The vertical position *y* on the sample is estimated from the vertical pixel number taking into account the pixel size (*P* = 23.5 μm) and magnification (*γ* = −0.3). For comparison, the results of the homogeneous samples with pure lactose (*T*_1_(*p, y*)) and pure D-fructose (*T*_2_(*p, y*)) are shown in top and bottom of Fig. 6(b), respectively. Both homogeneous samples show an approximately uniform image in the vertical direction. Compared to the top and the bottom, Fig. 6(b) middle has a boundary near pixel number 100, where the upper pixels are lactose and the lower pixels are D-fructose. This feature is in good agreement with the optical image in Fig. 6(a).

The distribution of each molecule can be evaluated more quantitatively via independent component analysis^27^. In this method, the spectrum is assumed to be a linear combination of several different spectra and another unrelated noise spectrum. This analysis can be applied to the absorption spectra, which is defined by *A* = 1 − *T*, where *A* is the absorption and *T* is the transmittance. The position-dependent absorption spectrum *A(f, y*) at a position *y* is expressed as


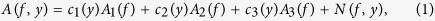


where *f* is the frequency, *A*_*i*_(*f*) is the absorption spectrum of each material indexed *i* (1: lactose, 2: fructose, and 3: frequency independent components), and *c*_*i*_(*y*) is the distribution coefficient of the material *i*, which is a function of the position *y*. Here, we assumed *A*_3_(*f*) = 1, which is primarily caused by the reflection of the aluminum frames and the Zeonex plates. *N(f, y*) is the noise component, which has no correlation with the other materials. The coefficients *c*_1_(*y*) and *c*_2_(*y*) indicate how lactose and D-fructose, respectively, are distributed in the sample.

To apply the analysis of Eq. (1), the pixel position *p* of each absorption image *A(p, y*) needs to be converted to frequency, *f*, using the calibration procedure described above. In the ideal case, *A*_*i*_(*p*) will not depend on the position *y* if the calibration curves, *f(p*), are perfectly uniform in the *y*-direction. However, in practice, the uniformity is not perfect, which can be seen as a distortion in the obtained images of the pure samples shown in Fig. 6(c) and (d). Therefore, a calibration curve needs to be determined for each position *y*, which is unsuitable for real time imaging because of the time-consuming nature of the analysis (for reference, the results of the frequency calibration are shown in Supplementary Information). However, such a calibration analysis can be avoided by changing the variable from *f* to *p* depending on the position *y*:


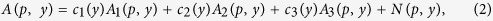


where *A*_*i*_(*p, y*) is the absorption image of the pure material indexed *i* and *N(p, y*) is the noise component. Compared to Eq. (1), the absorption image of a pure sample *A*_*i*_(*f*) changes to *A*_*i*_(*p, y*) due to the non-uniformity of the calibration curves in the *y*-direction. In this way, the frequency calibration is avoided in the analysis. The scheme to determine the coefficients is described in “Independent component analysis” in “Methods.”

The analyzed coefficients *c*_1_(*y*) and *c*_2_(*y*) are shown in Fig. 6(c). Lactose (*c*_1_(*y*)) exists in the upper section while D-fructose (*c*_2_(*y*)) exists in the lower section, which is consistent with the position of the boundary shown in Fig. 6(a). The independent component analysis can also be performed simultaneously with data acquisition using a commercially available computer because all the calculations are linear operations. One stripe of Fig. 6(c) can be obtained as quickly as 15 fps. The stripe can contain 240 pixel which is the vertical pixel number of the THz camera.

## Discussion

**Figure 7:**
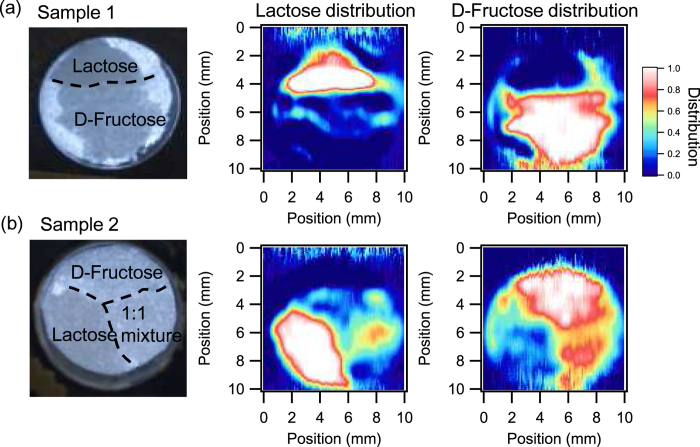
Molecule-specific spectroscopic two-dimensional images with one-dimensional sample scanning. (**a**) Test sample 1 and (**b**) Test sample 2 (left: optical image, middle: lactose distribution, and right: D-fructose distribution).

We demonstrated real-time spectroscopic imaging in 1D space. Due to this real-time feature, a 2D image of the molecular distribution can be obtained in short acquisition times by scanning the sample in only one-dimension. The image of the spatial distributions of lactose and D-fructose are obtained as shown in Fig. 7(a), the two molecules are clearly distinguished, and the obtained image agrees well with the optical image. These images consist of 100-stripe forms. Each image required approximately 40 seconds. The imaging speed was limited by the speed of the motor for sample scanning. If we assume the maximum frame rate (15 fps), the minimum acquisition time is 6.7 seconds. By optimizing the scanning systems, the acquisition time should approach that of theoretical limits.

Furthermore, the independent component analysis can be applied to a mixture of the molecules. To demonstrate this capability, another test sample was prepared as shown in Fig. 7(b). In this sample, there were three domains: pure lactose, pure D-fructose, and a half-and-half mass ratio mixture of lactose and D-fructose. Following the same procedure, a molecule-specific image was obtained. The mixture domain appears in both the lactose distribution and the D-fructose distribution. It is seen that the distribution of the two molecules in the mixture domain is clearly distinguished in the THz imaging even though it is difficult to distinguish them in the optical image.

In this study, a high-power THz source from a regenerative amplifier laser system is used to obtain high signal to noise ratio, considering sensitivity THz camera in the state-of-the-art. However, our method can be applied with incoherent THz sources if the THz camera has high enough sensitivity. Considering the recent rapid improvements of THz camera sensitivity, such a THz camera should be developed in near future, and our imaging technique will be wide spread with simpler light sources such as thermal light sources. In addition, using an uncooled THz camera has advantage for several applications where it is difficult to use coolants.

In conclusion, we demonstrated THz real-time spectroscopic imaging using a high-sensitivity uncooled THz camera. A broadband multi-channel spectrometer near 1 THz was constructed with reflection type diffraction grating and a high-power broadband THz source, and was expanded to imaging spectroscopy exploiting the two-dimensionality of the detector. Molecule-specific imaging, at real-time for 1D images and high-speed for 2D images, were demonstrated taking an advantage of the fingerprint spectra in the THz region. Such molecule-specific imaging could be applied to observe the transfer of specific bio-molecules or chemical reaction processes. The short-time acquisition of 2D images with 1D sample scanning has various potential applications such as security, biomedicine, or semiconductor wafer inspections.

## Methods

### Experimental setup

Figure 1 shows a schematic of the experimental setup. To achieve broadband and real-time spectroscopic imaging near 1 THz with high enough signal to noise ratio, three components are required: (1) a high-power and broadband THz source, (2) diffraction grating for the THz waves, and (3) a THz camera with high sensitivity for the THz spectroscopic measurement.

High-power broadband THz pulses were generated with optical rectification in a LiNbO_3_ crystal with tilted-pulse-front excitation^16^,^17^. A regenerative amplified Yb:KGW laser (PHAROS-15W, Light Conversion, Ltd.) with a repetition rate of 75 kHz, center wavelength of 1028 nm, pulse energy of 0.2 mJ, and pulse duration of 280 fs was used as a fundamental light source. A transmission grating with 1600 lines/mm was used for the tilted-pulse-front excitation, and the image of the grating was transferred with a lens pair whose focal lengths were 75 mm and 50 mm. The generated THz pulses had an average power of 3 mW, which is evaluated with a thermal power sensor for THz waves (3A-P-THz, Ophir Optronics Solutions, Ltd.). Fundamental light in the THz beam path is eliminated with a black polypropylene (BPP) filter, as shown in Fig. 1(b).

The diffraction grating for the THz waves was designed to work from 1.3 THz to 2.1 THz. In general, the spectral span of the diffraction grating spectrometer is limited to one octave due to second order diffraction. The lower frequency edge of the span is determined by Wood’s anomaly, and the higher is twice that frequency. To obtain high diffraction efficiency, the braze wavelength should be in the middle of the frequency span. Therefore, an aluminum diffraction grating with a periodicity of 253 μm and a braze angle of 18.4° was manufactured using a numerically controlled machine. In this design, Wood’s anomaly frequency is 1.19 THz, and twice that is 2.38 THz, covering the frequency span of interest. The braze wavelength corresponds to 1.73 THz, which is approximately in the middle of the spectral span. The diffraction efficiency was also numerically calculated using commercial software (DiffractMOD, Synopsys, Inc) with a rigorous coupled-wave analysis method. Below 2 THz, the p-polarization has higher efficiency and a flatter spectrum than the s-polarization (See the Supplementary material).

A high-sensitivity THz camera (IRV-T0831HS, NEC) was used as the detector^21^. The camera had a frame rate of 30 fps and a lock-in detection function to improve the signal to noise ratio (SNR). For the lock-in detection, the fundamental beam for the THz generation was modulated using an optical chopper with a frequency of 15 Hz, which was synchronized with half the frame rate of the camera. The camera had 320 × 240 pixels, and the pixel size was 23.5 μm × 23.5 μm. Because this pixel size is much smaller than the wavelength of the THz wave, spatial integration and smoothing with finite pixel numbers (typically 8 × 8 pixels or 12 × 12 pixels) were effective to improve the SNR without losing spatial resolution. Averaging several frames over time is also an effective way to improve the SNR. The lock-in detection and averaging in pixels and frames were processed simultaneously with the image acquisition, and the images could be refreshed in 15 fps.

The entire experimental setup is shown in Fig. 1(b), which is the combination of these three components. We used commercially available THz lenses (Tsurupica, Pax Corp.). The generated THz beam from the LiNbO_3_ crystal was collimated using a THz lens (f = 100 mm) that goes through the sample and is focused by another lens (f = 50 mm) to an iris that is placed at the entrance aperture of the THz spectrometer. Prior to the sample, the polarization of the THz beam was converted from horizontal to vertical via a periscope configuration that consisted of two 45° reflection mirrors resulting in a p-polarization incidence on the THz grating. This process also changed the height of the THz beam and the in-plane propagation direction by 90°. The lens pair with a focal length of 30 mm resulted in a frequency-resolved image of the entrance aperture in the horizontal direction using the THz grating placed between the lens pair. The frequency-resolved image was reductively projected to the sensor plane of the THz camera with the lens pair whose focal lengths were 100 mm and 30 mm. To eliminate effects of the absorption of water vapor, the entire setup was purged with dry air.

### TDS measurements

For the comparison, THz TDS measurements were also performed. A regenerative amplified Ti:Sapphire laser (RegA9000, Coherent Inc.) with a repetition rate of 120 kHz, center wavelength of 800 nm, pulse energy of 6 μJ, and pulse duration of 110 fs was used as a fundamental light source. THz pulses were generated with optical rectification in a LiNbO_3_ crystal with tilted-pulse-front excitation. THz electric field waveforms were measured by electro-optic sampling with a 1-mm-thick ZnTe(110) crystal. To eliminate effects of the absorption of water vapor, the entire setup was purged with dry air or dry nitrogen gas.

### Sample preparation

In order to confirm the validity of our measurement scheme, we used some test samples: bandpass filters and sugar samples.

The bandpass filters are commercially available ones (BP-13 N1, BP-16 N1, and BP-19M, CDP corp.) which are based on frequency selective surfaces of metallized microstructures. The filters consist of multiplex (non-interference) structures implemented in three- or four-layer configurations^28^.

The sugar samples were prepared by pounding the powder with a mortar, and sandwiched by hand between two 2-mm-thick window plates (ZEONEX, Zeon Corp.), transparent in both the visible and THz regions. An aluminum spacer was inserted between the plates to define the thickness of the powder samples.

Note that the Fabry-Perrot interference of the sugar samples were not observed because of the small mismatch of the refractive indices of the sample and the window. The Fabry-Perrot interference of the window plates were also not observed because the free spectral range (~0.025 THz) was much smaller than the frequency resolution of the spectrometer.

In Fig. 4, there are still small difference between the absolute values of transmittance because the sugar samples we measured by TDS and camera spectrometer were prepared independently, thus not the exactly same samples. The thickness of the lactose sample for TDS was 100 μm, which was slightly different from the samples used for the imaging experiment. The difference of the amount of the powders may lead to the discrepancy of these two spectra. However, the observed absorption frequency is exactly the same, 1.37 THz in Lactose and 1.72 THz in D-Fructose. This match is important in our demonstration of molecule-specific imaging. In addition, the agreement between absolute values of the transmission spectra obtained by TDS and camera spectrometer was confirmed by the measurements of bandpass filters, shown in Fig. 3(e).

### Calibration of the frequency

The relationship between the pixel position *p* and the frequency of the THz wave *f* satisfies the equation of diffraction:


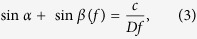


where *α* is an incident angle, *β(f*) is a frequency-dependent diffraction angle, *c* is the speed of light, and *D* is the periodicity of the grating (*D* = 253 *μ*m). As shown in Fig. 3(a), the pixel position *p(f*), where frequency *f* component is focused, satisfies the equations:


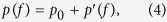



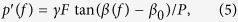


where *p*_0_ is the position of the center pixel of the camera (*p*_0_ = 160), *γ* is the magnification factor of the lens pair between the Fourier plane and the sensor plane (*γ* = −0.3 in our case), *F* is the focal length of the lens before the Fourier plane (*F* = 30 mm), *β*_0_ is the central angle of diffraction, and *P* is the pixel size of the camera (*P* = 23.5 *μ*m). In addition, the systematic error from the aberration was taken into account, and Eq. (4) is replaced with


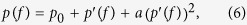


where *a* is a coefficient of distortion. The inverse function of Eq. (6) is defined as *f(p*).

The absorption spectrum of water vapor, which is available in a database^26^, is expressed by the absorption line frequencies *f*_*i*_ and the oscillation strengths *A*_*i*_, as shown in Fig. 3(b). Because the spectrometer has finite frequency resolution and the THz wave has a broadband spectrum, a frequency-dependent resolution function needs to be considered. Assuming that the resolution function is Gaussian, the fitting transmittance spectrum *T*_fit_(*p*) from the database can be written as


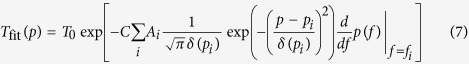


where *T*_0_ is a constant, *C* is the optical density, which depends on the humidity or the path length, *p*_*i*_ = *p(f*_*i*_) is the pixel position of the *i*-th absorption line, and *δ(p*) is the frequency-dependent resolution pixel width. Because higher frequency components can be focused tighter, we assumed that the pixel number that corresponds to the frequency resolution for higher frequencies becomes smaller, and the test function for the resolution *δ(q*) is assumed to be


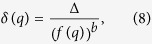


where *Δ* and *b* are constants. In this fitting, there are seven free parameters: *α, β*_0_, *a, Δ, b, T*_0_, and *C.* Using the simulated annealing method^29^, ∑_*p*_|*T(p*) − *T*_fit_(*p*)|^2^ in the domain of interest can be minimized and the parameters can be determined, as shown in Table 1.

Note that other materials with well-known spectra can be also used for this calibration. For example, a Fabry-Perrot cavity of a bare silicon wafer with thickness of 350 μm can be used because it has a free spectral range of 0.125 THz, which matches the frequency range of the spectrometer.

### Independent component analysis

To determine the coefficients of the material distribution (*c*_*i*_(*y*)), the inner products are defined as


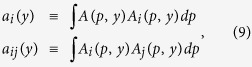


and the noise component *N(p, y*) is assumed to satisfy


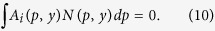


Then, the relationships between the coefficients and the inner products are


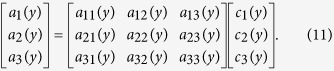


Using the inverse matrix, the coefficients can be determined to be


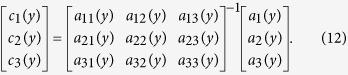


## Additional Information

**How to cite this article:** Kanda, N. *et al*. Real-time broadband terahertz spectroscopic imaging by using a high-sensitivity terahertz camera. *Sci. Rep.* **7**, 42540; doi: 10.1038/srep42540 (2017).

**Publisher's note:** Springer Nature remains neutral with regard to jurisdictional claims in published maps and institutional affiliations.

## Supplementary Material

Supplementary Information

## Figures and Tables

**Table 1:** Determined parameters in the fitting for frequency calibration.

*α* (deg.)	*β*_0_ (deg.)	*a*	Δ	*b*	*T*_0_	*C*
1.60	49.0	−8.30 × 10^−5^	22.7	1.19	0.958	0.0314
