# Supplementary material for: Real-time broadband terahertz spectroscopic imaging by using a high-sensitivity terahertz camera
Source: Sci Rep. 2017 Feb 15;7:42540. doi: 10.1038/srep42540 (PMC5309736; doi:10.1038/srep42540)
Supplement: Supplementary Information [file srep42540-s1.pdf]

Supplementary information

Real-time broadband terahertz spectroscopic imaging by using a high-sensitivity terahertz camera

Natsuki Kanda[1,2], Kuniaki Konishi[3], Natsuki Nemoto[4], Katsumi Midorikawa[1,3*], and Makoto Kuwata-Gonokami[3,4*]

[1]*RIKEN Center for Advanced Photonics, RIKEN, 2-1 Hirosawa, Wako, Saitama 351-0198, Japan*

[2]*Photon Science Center, The University of Tokyo, 7-3-1 Hongo, Bunkyo-ku, Tokyo 113-8656, Japan*

[3]*Institute for Photon Science and Technology, The University of Tokyo, 7-3-1 Hongo, Bunkyo-ku, Tokyo 113-0033, Japan*

[4]*Department of Physics, The University of Tokyo, 7-3-1 Hongo, Bunkyo-ku, Tokyo 113-0033, Japan*

[*]*Correspondence and requests for materials should be addressed to K.M. (email: kmidori@riken.jp) or to M.K.-G. (email: gonokami@phys.s.u-tokyo.ac.jp).*

Simulation of the signal efficiency in the spectrometer

To estimate the efficiency of the THz light in the entire spectrometer system, we calculated the signal density spectra. The diffraction efficiency was numerically calculated using commercial software (DiffractMOD, Rsoft Design Group, Inc) with a rigorous coupled-wave analysis method. The results are shown in Fig. S1(a). Below 2 THz, the p-polarization has higher efficiency and flatter spectrum than the s-polarization. The signal density spectra were estimated from the products of the spectrum of the light source ($I(f)$), diffraction efficiency ($\eta_p(f)$ or $\eta_s(f)$, depending on the polarization), and sensitivity of the camera ($\eta_c(f)$). The spectrum of the THz source was assumed to have a 1 THz center frequency and a 1 THz full width at half maximum (FWHM) bandwidth (Fig. S1(b)). The frequency dependent sensitivity of the THz camera is also plotted in Fig. S1(b) [1], and their products for the p- and s-polarizations are shown in Fig. S1(c). The efficiencies of the total signal count are estimated to be 20% for p-polarization and 11% for s-polarization. Note that the component higher than 2.4 THz is quite small compared to the lower frequency components in this case. Therefore, second order diffraction can be ignored here. If the second order component affects the spectroscopic measurement with other series of THz sources, gratings, and detectors, a low-frequency pass filter should be inserted.

References

[1] Nemoto, N., Kanda, N., Imai, R., Konishi, K., Miyoshi, M., Kurashina, S., Sasaki, T., Oda, N. & Kuwata-Gonokami, M. “High-sensitivity and broadband, real-time terahertz camera incorporating micro-bolometer array with resonant cavity structure,” IEEE Trans. THz Sci. Technol., **6**, 175-182 (2016).

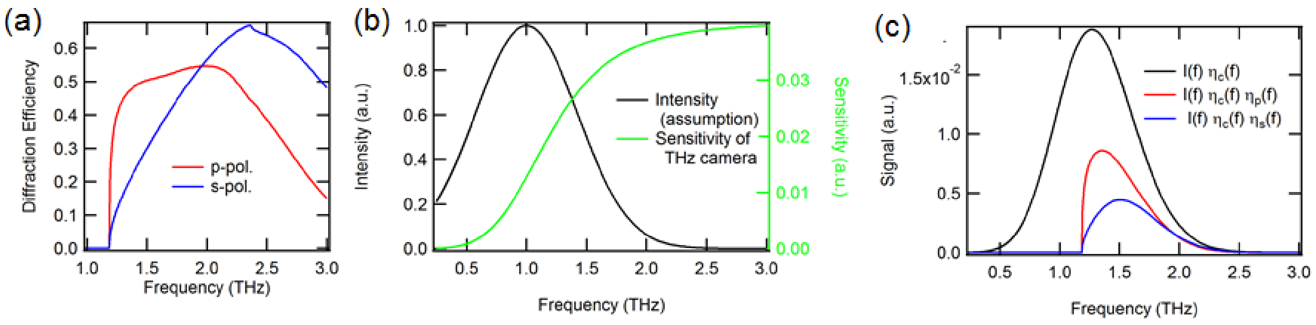

Fig. S1. (a) The simulation results of the diffraction efficiency of the brazed grating for p-polarization (red) and s-polarization (blue). (b) The assumed THz spectrum with a center frequency of 1 THz and a FWHM of 1 THz (black) and the frequency-dependent sensitivity of the THz camera (green). (c) The estimate of the signal density spectra. The black curve is the product of the THz spectrum and the sensitivity of the camera, which are shown in (c). By multiplying the diffraction efficiency, the signal density spectra can be estimated for the p- and s-polarizations.

Frequency calibration for imaging spectroscopy

In imaging spectroscopy, frequency calibration can be performed by the same procedure (by measuring the transmittance of water vapor), described in Fig. 3. However, the calibration fitting should be repeated for each line of the vertical axis. The transmission image of water vapor is shown in Fig. S2(a), and calibrated image is shown in Fig. S2(b). To construct the calibrated image, interpolate analysis in frequency axis of the necessary. The distortion observed in the vertical direction in Fig. S2(a) is modified in Fig. S2(b) by this calibration.

The determined relationships between pixel and frequency are applied to the data in Fig. 6(b). Fig. S3(a) is the same data as Fig. 6(b). Calibrated images are shown in Fig. S3(b). The absorption lines at 1.37 THz for lactose and at 1.72 THz for D-fructose are clearly observed, and the frequency is completely reproduced. Note that, as discussed in the “Spectroscopic imaging” part in the “Results” section, this frequency calibration is not necessary for molecule specific imaging, because independent component analysis can be applied to the data even before the frequency calibration. This is preferable

for achieving real-time spectroscopic imaging because the time-consuming fitting procedures and interpolate analysis can be avoided.

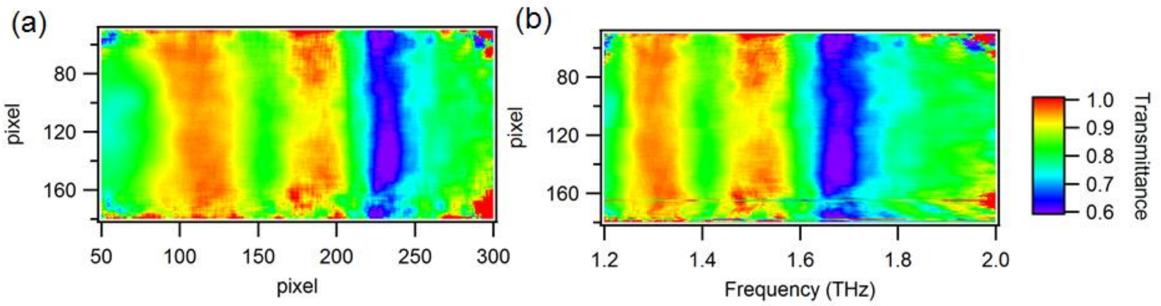

Fig. S2. (a) The transmittance image of water vapor, which is used for frequency calibration. (b) The reconstructed image of transmittance of water vapor in frequency axis.

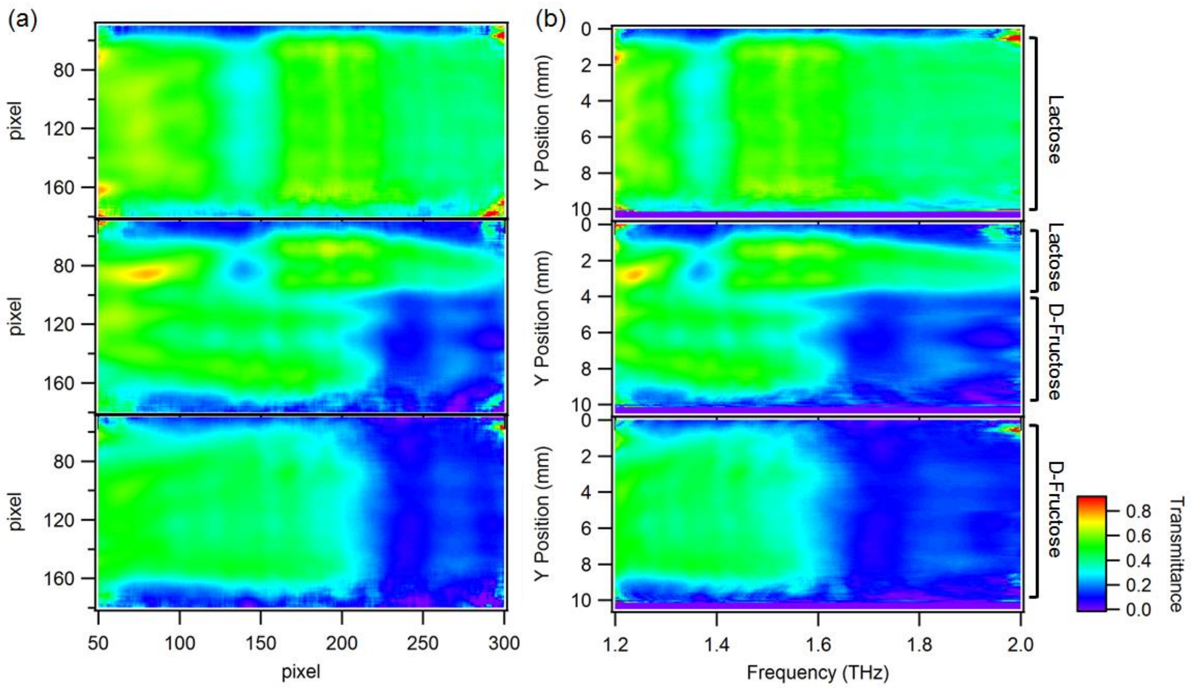

Fig. S3. (a) Transmittance images of test sample 1 and pure lactose, fructose, which are also shown in Fig. 6(b). (b) Calibrated images.
